# Urinary Tract Infection as a Potential Cause of Urethral Obstruction in Patients with an Indwelling Allium® Bulbar Urethral Stent

**DOI:** 10.5152/tud.2026.26004

**Published:** 2026-05-15

**Authors:** Ufuk Yavuz, Seyfettin Çiftçi, Engin Telli, Tijen Nemut, Mustafa Melih Çulha

**Affiliations:** 1 Department of Urology, Karamanoğlu Mehmetbey University School of Medicine, Karaman, Türkiye; 2 Department of Urology, Aktif International Hospital, Kocaeli, Türkiye; 3 Department of Urology, Kocaeli University School of Medicine, Kocaeli, Türkiye; 4 Department of Nursing, Institute of Health Science, Sakarya University, Sakarya, Türkiye

**Keywords:** Urethral obstruction, urethral stricture, urinary tract infection

## Abstract

**Objective:**

Urethral obstruction is a clinically relevant complication of retrievable urethral stents. This study aimed to evaluate the association between stent-related urinary tract infection (UTI) and urethral obstruction in patients treated with an Allium® bulbar urethral stent (BUS).

**Methods:**

Ninety-nine patients who underwent Allium® BUS placement for recurrent bulbar urethral stricture were retrospectively analyzed. Patients were followed monthly with uroflowmetry, postvoid residual urine measurement, and quantitative urine culture. In cases of stent removal, both urine and stent cultures were obtained and analyzed.

**Results:**

Premature stent removal due to urethral obstruction was required in 14 of 99 patients (14.1%). Urine cultures were positive in 12 of these 14 patients (85.7%), with *Escherichia coli*, *Klebsiella pneumoniae*, and *Enterococcus faecalis* being the most frequently isolated organisms. Stent cultures were positive in 12 of these 14 patients (85.7%), demonstrating similar pathogens, including *Enterococcus faecalis*, *Klebsiella pneumoniae*, and *Proteus mirabilis*. In patients whose stents were removed on time or due to other complications without obstruction, positive urine and stent cultures were observed in 6.1% and 1.5% of cases, respectively. The rate of positive urine cultures was significantly higher in patients with urethral obstruction (*P* < .01).

**Conclusion:**

The findings suggest that urethral obstruction in patients with an indwelling Allium® BUS is more likely related to UTI rather than mechanical stent failure.

**Main Points**

- Urethral obstruction in the Allium® bulbar urethral stent is primarily driven by infection and bacterial colonization rather than mechanical failure.
- A strong correlation exists between obstruction and infection, with 85.7% of obstructed patients demonstrating positive urine and stent cultures.
- Positive culture rates are significantly higher in patients with urethral obstruction compared to those without obstruction (*P* < .01).
- Bacterial colonization and biofilm formation are the principal triggers for luminal occlusion, despite the stent’s protective polymeric coating.
- Standard antibiotic prophylaxis may be insufficient to prevent stent colonization and the subsequent need for premature removal.

## Introduction

Urethral stricture disease, resulting from fibrosis of the urethral mucosa and surrounding tissues, represents a significant urological condition with an estimated incidence of 627 per 100,000 patients.^1^^,^^2^ Urethral dilation and visual internal urethrotomy (VIU) remain the most commonly used initial treatment options; however, their long-term success rates are limited, particularly in recurrent or long-segment strictures.^3^

Urethral stents have been proposed as a minimally invasive alternative to repeated VIU procedures.^4^ Since their introduction in the early 1990s, various temporary and permanent urethral stents have been developed, with reported long-term success rates of approximately 63% in patients with recurrent strictures. More recently, temporary placement of a novel bulbar urethral stent (BUS) has demonstrated success rates as high as 81%.^5^

Despite these favorable outcomes, stent-related complications remain a concern. Urethral obstruction is one of the most significant complications associated with retrievable stents, often necessitating premature removal. In urological practice, long-term indwelling stents are known to be limited by infection and encrustation, which may contribute to stent malfunction.^6^^,^^7^ The present study aimed to investigate the association between stent-related urinary tract infection (UTI) and urethral obstruction in patients treated with an Allium® BUS.

## Material and Methods

Between March 2011 and February 2021, 99 men treated with an Allium® BUS for recurrent benign bulbar urethral stricture were retrospectively evaluated. Patients with strictures involving other segments of the urethra were excluded. Indications for BUS placement included recurrent bulbar urethral strictures requiring at least 1 prior VIU. All patients provided written informed consent prior to stent implantation.

Ethics approval was obtained from the Kocaeli University Ethics Committee for Non-Interventional Clinical Research Ethics Committee (Decision No: *GOKAEK-2021/5.24-2021/5.24*, Project No: *2021/93*). The study was approved on March 4, 2021, and conducted in accordance with the principles of the Declaration of Helsinki and relevant national regulations.

The etiologies of urethral stricture included trauma in 45 patients, previous surgery in 36 patients, infection in 9 patients, and unknown causes in 9 patients. Preoperative urine cultures were obtained in all patients. Eighteen patients had positive urine cultures (>100,000 CFU/mL), including *Klebsiella* spp., extended-spectrum beta-lactamase–producing *Escherichia coli*, and *Enterococcus faecalis*. These patients received culture-directed antibiotic therapy and underwent stent placement only after achieving negative urine cultures. Patients with negative preoperative urine cultures received prophylactic antibiotic therapy with levofloxacin starting 1 day before stent placement and continuing for 10 days postoperatively.^6^^,^^8^

The Allium® BUS is a fully covered, self-expandable, large-caliber metallic stent designed specifically for bulbar urethral strictures. The stent consists of a coiled super-elastic metal framework covered with a polymeric coating intended to prevent mucosal hyperplasia, reduce bacterial adherence, and minimize encrustation and calcification within the stent lumen.^6^^,^^9^

All procedures were performed under general or spinal anesthesia with the patient in the lithotomy position. Internal urethrotomy was performed prior to stent deployment in all cases. Stricture length was documented, and an appropriately sized BUS was inserted distal to the external urethral sphincter using a dedicated delivery system.

The planned indwelling time was 12 months. Patients were followed monthly with uroflowmetry, ultrasonography to assess postvoid residual urine volume, and quantitative urine cultures. Urethral obstruction was defined as impaired urinary flow requiring premature stent removal. Removed stents were cultured to evaluate bacterial colonization.

Removed stents were placed in sterile conical centrifuge tubes containing 10 mL of Mueller–Hinton broth and vortexed for 5 minutes. Serial dilutions were cultured on sheep blood agar plates and incubated for 18-24 hours. Growth of a single bacterial species exceeding 100,000 Colony Forming Unit (CFU)/mL in patients presenting with clinical symptoms of UTI (e.g., dysuria or pelvic discomfort) was considered positive. For the purposes of this study, cases of asymptomatic bacteriuria were not classified as positive urine cultures. Microbial identification was performed using the VITEK® 2 automated system.^9^^,^^10^

### Statistical Analysis

Statistical analyses were performed using Statistical Package for the Social Sciences (SPSS) Statistics for Windows, Version 21.0 (IBM Corp., Armonk, NY). The Mann–Whitney *U* test was used to compare independent variables. A *P* value < .01 was considered statistically significant. Data are presented as median (interquartile range).

## Results

The median age of patients with urethral obstruction was 58 years (IQR 35-69), compared with 67 years (IQR 34-72) in non-obstructed patients. Patient characteristics are summarized in Table 1. The rate of positive urine cultures was significantly higher in patients with urethral obstruction (*P* < .01). The indwelling time of stents was significantly shorter in patients who developed obstruction (*P* < .01).

Fourteen of 99 patients (14.1%) experienced impaired urinary flow due to stent obstruction and underwent premature stent removal. Twelve of these 14 patients (85.7%) had positive urine cultures, and eleven (78.5%) had positive stent cultures (Table 2). Identified microorganisms included *Klebsiella pneumoniae*, Extended-Spectrum Beta-Lactamase (ESBL)-positive *E. coli*, *Enterococcus faecalis*, and *Proteus mirabilis*. During the systematic urethroscopic evaluation performed during the removal procedure, it was observed that the stents remained in their original positions in all 14 cases, and no signs of proximal or distal displacement were noted. Significant intraluminal crusting and calcification were present, accompanied by visibly cloudy urine. Although minor, non-obstructive reactive changes were observed in the surrounding urethral tissue in the majority of patients, no intraluminal tissue ingrowth, mucosal hyperplasia, or obstructive granulation tissue was identified within the stent lumen.

Among patients whose stents were removed at the planned time or prematurely for reasons other than obstruction, positive urine cultures were observed in 6 patients (6.06%), and positive stent cultures in 2 patients (2%). No major complications were observed, except for early stent migration in 3 patients (3%).

## Discussion

Urethral and ureteral stents are widely used in urological practice, and BUS have been employed since the late 1980s.^5^ Previous studies have demonstrated an increased risk of stent obstruction in patients with concurrent UTI.^11^

Bacterial colonization plays a central role in stent-associated UTIs and may progress to encrustation and mechanical obstruction.^8^^,^^12^^,^^13^ Biofilm formation on stent surfaces protects bacteria from antibiotic therapy, meaning that negative urine cultures do not necessarily exclude stent colonization.^9^

Mechanical stent occlusion remains one of the most common complications requiring stent removal. Bacterial adherence, biofilm formation, and subsequent mineral deposition may lead to encrustation and luminal obstruction.^14^ A similar mechanism likely contributes to obstruction of the Allium® BUS despite its polymeric coating.

Accordingly, Çulha et al. reported that stent obstruction occurred in 6 out of 54 patients who underwent Allium® BUS for urethral stricture, due to migration and in 4 due to infection. This shows that infection is a notable cause of obstruction in non-migrating temporary urethral stents.^5^ In patients with urethral stricture, a chronic UTI that existed before stent placement and could not be fully resolved with treatment may have subsequently led to stent infection.

The Allium® BUS is designed to prevent mucosal hyperplasia and maintain urethral patency during a planned indwelling period of up to 12 months.^5^ However, the presence of a foreign body may facilitate bacterial colonization, ultimately necessitating stent removal.^15^ Even when urine cultures are sterilized with antibiotics, stent colonization may persist and predispose patients to recurrent infection and obstruction.^9^^,^^10^

Colonization rates of indwelling ureteral stents range from 42% to 90%, with only a subset of patients developing symptomatic UTI.^16^^-18^ Longer indwelling times increase this risk. Additionally, infections caused by urease-positive organisms such as *Proteus mirabilis* may accelerate encrustation by increasing urinary alkalinity.^14^^,^^19^

Jordan et al. reported no stent occlusion with the Memokath® thermo-expandable urethral stent despite a high rate of bacteriuria.^20^ In contrast, the study demonstrated a strong association between infection and obstruction in patients with Allium® BUS, suggesting that infection-related mechanisms may play a more prominent role in stent malfunction.

Although the relationship between infection and obstruction is not definitive in patients who have undergone urethral stent placement, encrustation and cloudy appearance in stents removed prematurely due to obstruction and positive culture results from these stents strengthen this association. Furthermore, the identification of postoperative infection-related obstructions in ureteral stents, as reported in the literature, supports the findings.^11^

To the best of knowledge, data addressing infection-related obstruction of BUS remains limited. The findings indicate that prophylactic antibiotic therapy alone may not be sufficient to prevent stent colonization and subsequent obstruction.

The limitations of this study include its retrospective design and relatively small sample size. Nevertheless, the consistent association between urethral obstruction and stent-related infection supports the clinical relevance of the findings.

In conclusion, the Allium® BUS demonstrates good anatomical adaptation and effectively prevents mucosal hyperplasia due to its polymeric coating. However, UTI appears to be a major contributing factor to urethral obstruction in patients with an indwelling Allium® BUS. Improved strategies to prevent bacterial colonization and encrustation may enhance stent performance and clinical outcomes.

## Figures and Tables

**Table 1.** Characteristics of Patients with and without Urethral Obstruction after Allium® BUS Placement

	**Obstructed (n = 14)**	**Non-Obstructed (n = 85)**
Indwelling time, weeks (median, IQR)	12 (9-15)	27 (17-47)
Positive urine culture, n (%)	12 (85.7)	4 (4.7)
Positive stent culture, n (%)	11 (78.5)	1 (1.2)
Age, years (median, IQR)	58 (35-69)	67 (34-72)

BUS, bulbar urethral stent; IQR, interquartile range.

**Table 2.** Microbiological Findings in Patients with Urethral Obstruction (n = 14)

	**Urine Culture, n (%)**	**Stent Culture, n (%)**
Positive	12 (85.7)	11 (78.5)
Negative	2 (14.3)	3 (21.5)
